# Cortical organization restored by cochlear implantation in young children with single sided deafness

**DOI:** 10.1038/s41598-017-17129-z

**Published:** 2017-12-04

**Authors:** Melissa Jane Polonenko, Karen Ann Gordon, Sharon Lynn Cushing, Blake Croll Papsin

**Affiliations:** 1 Institute of Medical Sciences, The University of Toronto, Toronto, ON M5S 1A8 Canada; 2 Neurosciences and Mental Health, The Hospital for Sick Children, Toronto, ON M5G 1X8 Canada; 3 Department of Otolaryngology – Head & Neck Surgery, The University of Toronto, Toronto, ON M5G 2N2 Canada; 4 Otolaryngology – Head & Neck Surgery, The Hospital for Sick Children, Toronto, ON M5G 1X8 Canada

## Abstract

Early treatment of single sided deafness in children has been recommended to protect from neurodevelopmental preference for the better hearing ear and from social and educational deficits. A fairly homogeneous group of five young children (≤3.6 years of age) with normal right sided hearing who received a cochlear implant to treat deafness in their left ears were studied. Etiology of deafness was largely cytomegalovirus (*n* = 4); one child had an enlarged vestibular aqueduct. Multi-channel electroencephalography of cortical evoked activity was measured repeatedly over time at: 1) acute (0.5 ± 0.7 weeks); 2) early chronic (1.1 ± 0.2 months); and 3) chronic (5.8 ± 3.4 months) cochlear implant stimulation. Results indicated consistent responses from the normal right ear with marked changes in activity from the implanted left ear. Atypical distribution of peak amplitude activity from the implanted ear at acute stimulation marked abnormal lateralization of activity to the ipsilateral left auditory cortex and recruitment of extra-temporal areas including left frontal cortex. These abnormalities resolved with chronic implant use and contralateral aural preference emerged in both auditory cortices. These findings indicate that early implantation in young children with single sided deafness can rapidly restore bilateral auditory input to the cortex needed to improve binaural hearing.

## Introduction

There are significant consequences of single sided deafness (SSD) in childhood on auditory development and function^1–3^ but questions about treatment remain^4^. In the present study, we examined whether cochlear implantation of the deaf ear in a fairly homogeneous group of five young children with normal or near-normal hearing in the other ear can restore expected organization of the auditory cortices.

The prevalence of childhood unilateral hearing loss is estimated to be 0.06 to 3.0%^5,6^, and has known developmental and educational consequences^3,7,8^. These effects relate, in part, to poor spatial hearing^9,10^; indeed, unilateral listening in childhood reorganizes cortical areas involved in spatial awareness and attention^2,11,12^. Children with bilateral deafness who used one cochlear implant (CI) for some time prior to bilateral implantation are a unique group of single sided listeners who experience neurodevelopmental preference for the first/better hearing ear both in the brainstem^13–15^ and cortex^16^. Similar findings are reported from kittens born with unilateral deafness^17,18^ or experimentally induced unilateral/asymmetric hearing in young animals^19–21^. Such auditory asymmetries have consequences for processing binaural timing and level cues^19,22^ which are integral for locating and distinguishing one sound amongst many^23^. Importantly, bilateral cochlear implantation without delay protects from development of the “aural preference syndrome”^16^. Thus, to avoid problems of single sided listening and preserve opportunities for binaural hearing, it has been recommended that hearing loss (unilateral or bilateral) be treated by providing the most appropriate device in each ear as soon as possible^3^.

Despite research-based recommendations, treatment in children with unilateral hearing loss has been inconsistent^4^. In the case of SSD where an auditory nerve is present, a CI is arguably the most appropriate device to stimulate the impaired ear^24–26^ but this is not the present standard of care. Potential for success is suggested by benefits of electrical stimulation from a CI in one ear and amplified acoustic input through a hearing aid contralaterally (“bimodal” listening) in children with asymmetric hearing^27–30^ and from adults with SSD who were implanted to treat disruptive tinnitus in the deaf ear^24,26^. Benefits for listening to speech in noise are realized over listening with the unimplanted ear alone and increase as the duration of deafness decreases^25^. Early studies of implantation in children with SSD show early signs of benefit^9,10,31–34^ and one case report in an older child suggests the potential for longitudinal changes in crossmodal plasticity^35^. To understand the functional outcomes and define an optimal period for implantation in SSD, it is essential to address whether expected function in bilateral auditory pathways can be restored during early important developmental periods.

In the present study, plasticity of the neural input to auditory cortices was measured to assess whether expected representation can be restored by providing electrical stimulation from a CI in one ear with normal hearing in the other ear in early development. Results in a group of young children (≤3.6 years) who were deaf in their left ears from infancy demonstrate marked and rapid uptake of input from the newly implanted ear, restoring symmetric representation of both ears in the auditory brain.

## Results

**Figure 1:**
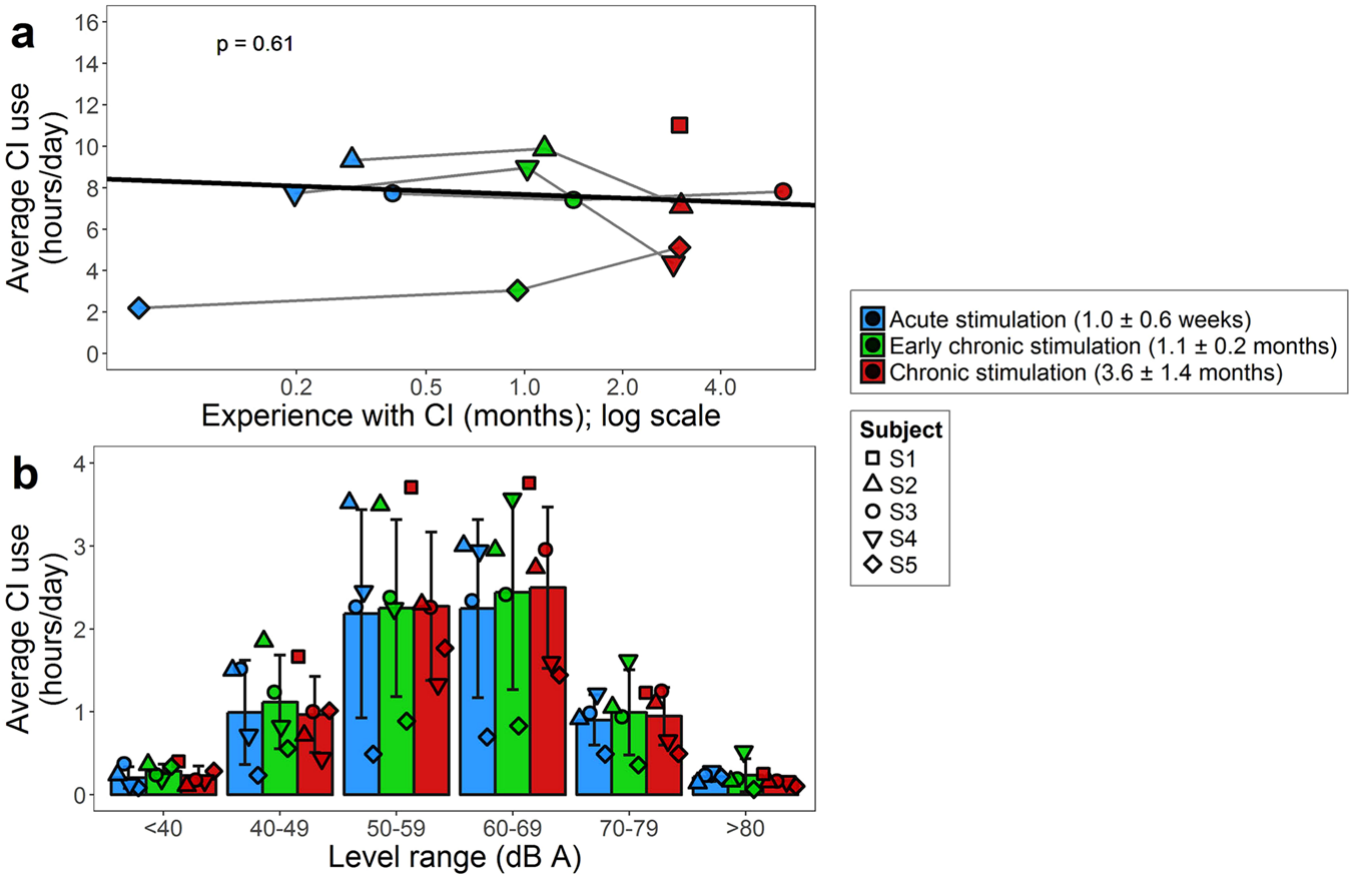
Evidence of chronic stimulation from datalogging information collected from the children’s cochlear implant processors. (**a**) The total average number of hours per day that each child used their cochlear implant (CI) is plotted against duration of CI experience (symbols connected with gray lines). Colours indicate the time points closest to the test time points that datalogs were collected, and the black line indicates the full linear mixed model based on *n* = 4 and log-transformation of CI use. (**b**) Average daily CI listening ± SD was predominantly at 50–69 dB A across time points. Datalogs were available for 4 children at all time points. A fifth child (S1) had one datalog at chronic stimulation (*n* = 5 at this time point). Data from four of the five children were also included in^36^.

All children were followed over their first six months of CI use. Daily use of the CI (mean ± SD = 7.1 ± 0.7 hours/day) was confirmed by datalogs available from the CI speech processor (Fig. 1a). One child (S5) showed few hours of average daily CI use at the first 2 test times; however, this child experienced frequent disconnections between the external and internal equipment (36.2 ± 11.0 times per day), as previously reported in young CI users^36,37^, accounting for an additional 5.4 ± 1.7 hours/day that the CI was worn. Daily CI use in the 4 children with complete data did not vary with CI experience (*χ* ^2^(1) = 0.3, *p* = 0.61). This time was mostly spent in environments with moderate sound levels (50–70 dB A) (level: *F*(5,15) = 18.6, *p* < 0.001) at all three time points (time: *F*(2,6) = 0.5, *p* = 0.62; level × time: *F*(10,30) = 0.2, *p* = 1.0) (Fig. 1b), consistent with datalogging information from a cohort of seven children with SSD^36^.

**Figure 2:**
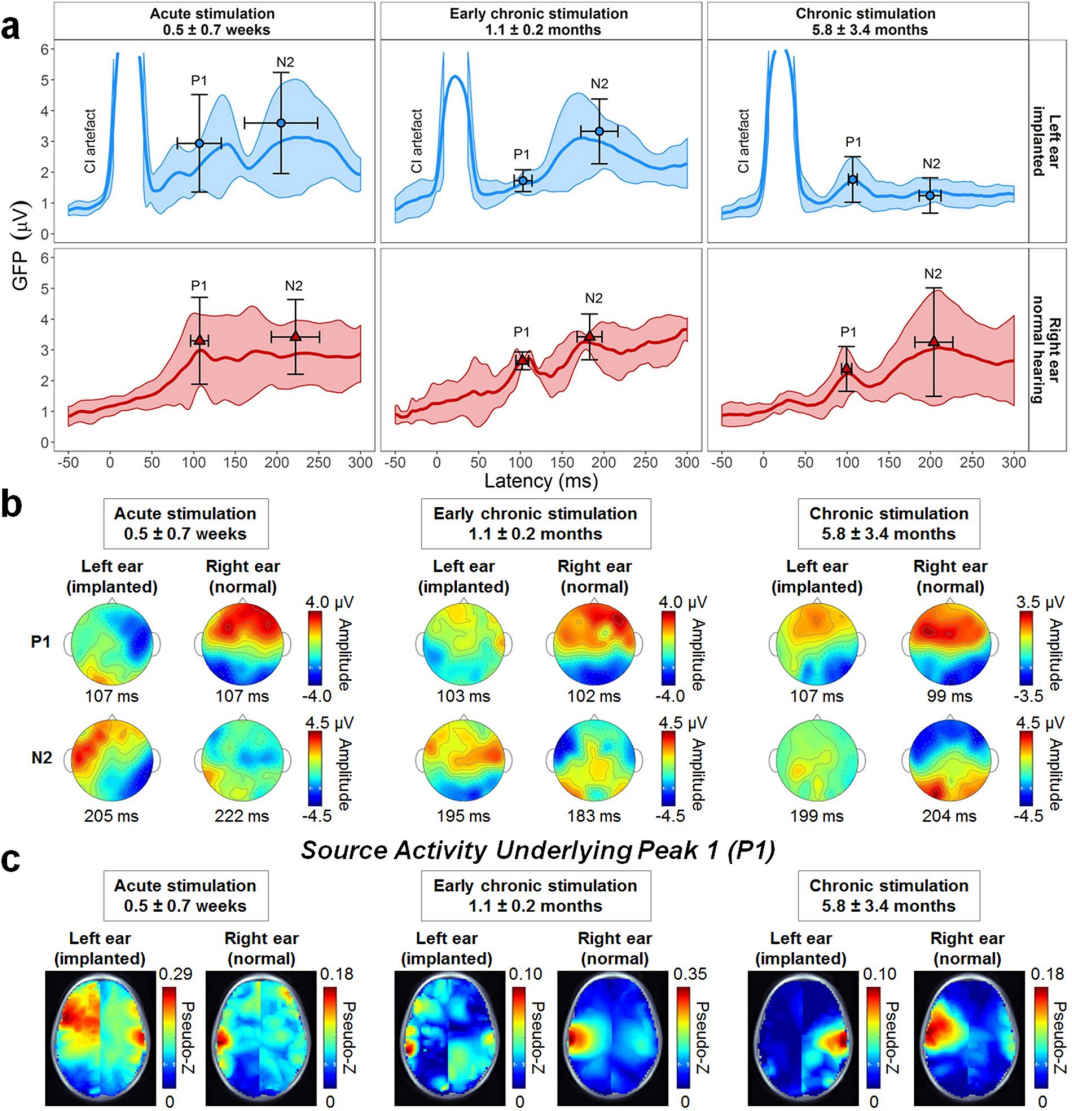
Surface recordings and source locations over time of CI stimulation: acute (initial activation week), early chronic (1 month), chronic (6 months). (**a**) Mean (solid line) ± SD (shaded region) global field power (GFP) as a function of post-stimulus time for each ear. The cochlear implant (CI) artefact is visible during stimulation presentation (0–36 ms), which occurred at latencies earlier than peaks P1 and N2. Mean ± 1SD of P1 and N2 peaks identified from each child’s GFP are indicated by symbols and errorbars. There were no significant changes (*p* > 0.05) in either peak amplitudes or latencies over time. (**b**) Topographical distributions of mean average-referenced surface responses at these mean peak latencies of P1 and N2. Opposite frontal-posterior polarities for P1 and N2 are evident for stimuli presented to the right normal hearing ear in all three recordings. Responses from the left ear CI were reversed in polarity at the first recording but the subsequent two recordings revealed frontal-positive activity for both P1 and N2. (**c**) Axial views of mean source activity in each of the 63,307 3 × 3 × 3 mm voxels (higher signal-to-noise pseudo-Z ratio in red) show widespread regions of activation underlying P1 for both the implanted and normal hearing ears upon acute stimulation. Activity became localized primarily to temporal lobes with chronic CI use. Because one child (S1) had missing data for the second visit, all measures for both P1 and N2 had *n* = 5 for acute and chronic stimulation; *n* = 4 for early chronic stimulation.

Longitudinal cortical recordings were successfully completed after CI activation. Two amplitude peaks (P1, N2) were identified in the mean global field power responses (Fig. 2a, mean ± 1SD latency and amplitude are indicated). Electrical artefact from the CI is clear during stimulus presentation (0 to 36 ms) in the left ear (blue) responses. There was no significant change in P1 or N2 amplitude (P1: time: *F*(2,22) = 0.3, *p* = 0.74; ear: *F*(1,22) = 0.3, *p* = 0.58; time × ear: *F*(2,22) = 0.2, *p* = 0.80; N2: time: *F*(2,22) = 1.9, *p* = 0.18; ear: *F*(1,22) = 0.2, *p* = 0.67; time × ear: *F*(2,22) = 0.6, *p* = 0.54) or latencies (P1: time: *F*(2,22) = 3.1, *p* = 0.07; ear: *F*(1,22) = 2.5, *p* = 0.13; time × ear: *F*(2,22) = 0.2, *p* = 0.84; N2: time: *F*(2,22) = 2.9, *p* = 0.08; ear: *F*(1,22) = 2.0, *p* = 0.17; time × ear: *F*(2,22) = 2.2, *p* = 0.14) over time for either the hearing or CI ear. Opposite polarities of P1 (frontal positive) and N2 (frontal negative) are largely consistent from the normal hearing ear in topographical plots over time (Fig. 2b). By contrast, the left CI evoked an abnormally frontal negative P1 and frontal positive N2 with acute stimulation which normalized at early chronic stimulation (Fig. 2b). Source activation for P1 evoked by the right normal hearing ear (Fig. 2c) indicated a consistent hotspot (high pseudo-Z signal-to-noise ratio in red) in the left temporal lobe at all times. Acute CI stimulation evoked a small region of activation in the right temporal cortex with high left frontal activity. At early chronic stimulation, this latter cortical response reduced with small hotspots of activity in both temporal lobes. With chronic CI exposure, activity became focused in the contralateral right temporal lobe.

**Figure 3:**
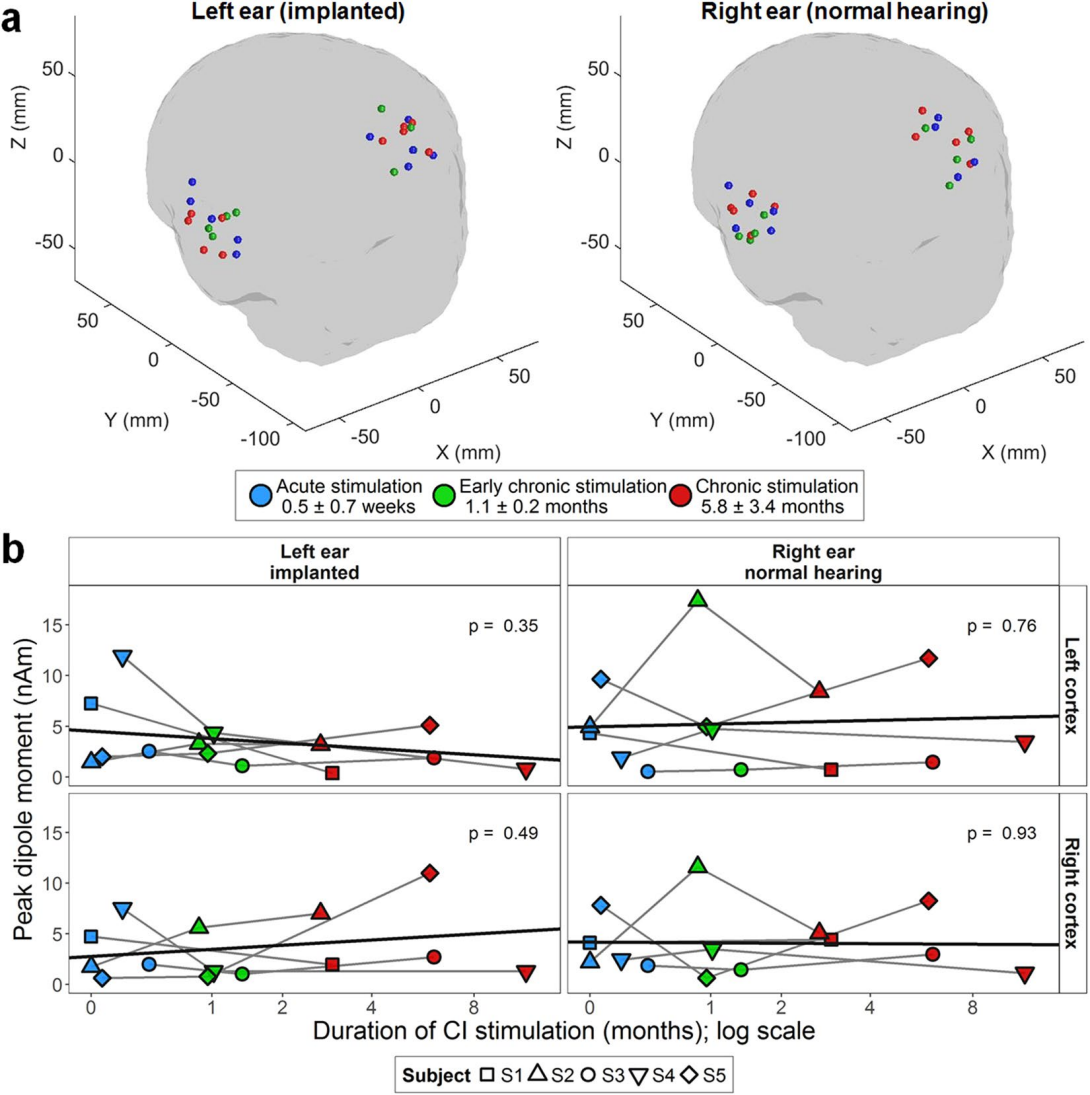
Peak dipole moments in the auditory cortices underlying P1. (**a**) Peak dipoles were located at similar locations for both the left implanted and right normal hearing ears over time. (**b**) Peak dipole moments for each ear and cortex individually varied somewhat with CI experience but there were no overall changes for right ear stimulation or left CI stimulation (*p* > 0.05). Symbols connected by gray lines indicate individual data, and the black line indicates the full linear mixed effects model using log-transformed CI use as a predictor. Data was missing from one child (S1) at early chronic stimulation, which had *n* = 4. Both acute and chronic stimulation time points had *n* = 5. Colours indicate test visits: acute (blue), early chronic (green), and chronic (red) CI stimulation.

Peak dipoles were measured from the voxels with the highest pseudo-Z in left and right auditory cortices (locations in Fig. 3a). Chosen voxels varied around the mean location for each cortex by 15.4 ± 5.5 mm and there was no significant change in voxel location over time (ear: *F*(1,7) = 0.0, *p* = 0.85; time: *F*(2,14) = 1.5, *p* = 0.25; *P* = 0.34; ear × time × coordinate: *F*(4,28) = 0.8, *p* = 0.45). Peak dipole moments did not significantly change with CI stimulation in either auditory cortex for either the normal right ear (Left Cortex: *χ* ^2^(1) = 0.09, *p* = 0.49; Right Cortex: *χ* ^2^(1) = 0.007, *p* = 0.93) or left CI ear (Left Cortex: *χ* ^2^(1) = 0.87, *p* = 0.35; Right Cortex: *χ* ^2^(1) = 0.48, *p* = 0.49).

**Figure 4:**
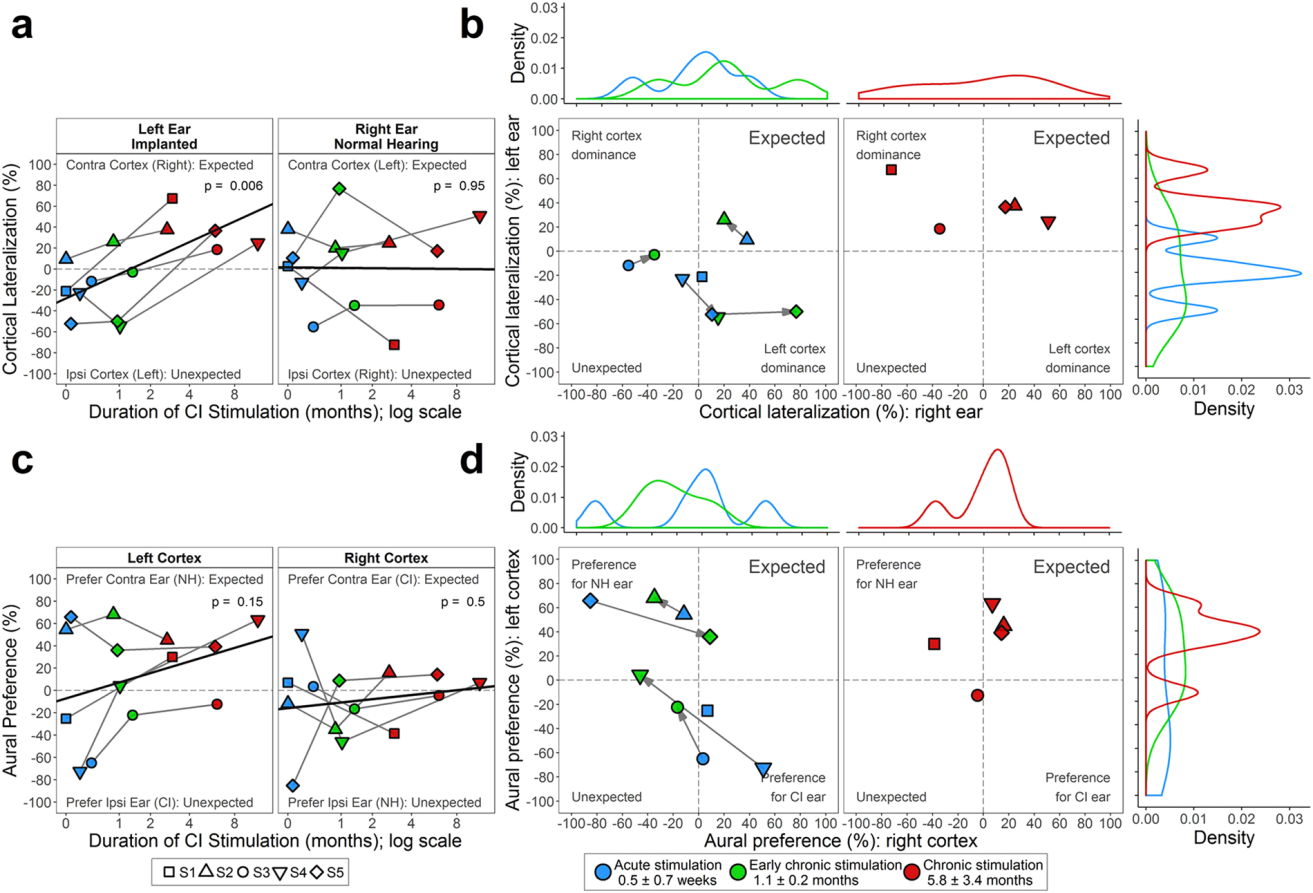
Abnormal cortical activity reverses with chronic CI stimulation. (**a**) Stimulation of the new left implanted ear revealed abnormal cortical lateralization (weighting) to the ipsilateral (left) cortex, which reversed towards the right cortex with chronic CI stimulation (*p* < 0.05). (**b**) Stimulation of the normal hearing ear revealed expected cortical lateralization to the contralateral left cortex in three of five children, which remained consistent with time. As a result, a distribution of expected cortical lateralization from both ears to the contralateral auditory cortex emerged after ~6 months. (**c**) Each cortex abnormally preferred stimulation from the ipsilateral ear in most children with acute stimulation, but tended to reverse towards preferring contralateral stimulation with CI stimulation (*p* < 0.05). (**d**) Both cortices preferred stimulation from only one ear at early time points. Distribution of preference for the expected contralateral ear emerged in both cortices by ~6 months.

Differences in dipoles between the left and right auditory cortices for each ear in each child were calculated as: Cortical Lateralization = 100 × [right cortex − left cortex]/[right cortex + left cortex]. Four children exhibited an unexpectedly large ipsilateral lateralization of cortical activity in response to the new left CI at acute stimulation and with early chronic CI use (Fig. 4a,b). A significant shift in lateralization to the expected contralateral right cortex was realized with chronic CI use in all children (*χ* ^2^(1) = 7.6, *p* = 0.006). Lateralization from the normal right ear was initially variable for the group (contralateral left (*n* = 2), bilateral (*n* = 2), and abnormal ipsilateral (*n* = 1)) but consistent for each child over time (*χ* ^2^(1) = 0.003, *p* = 0.96). Cortical lateralization from both ears, plotted for each child at initial CI use (two early time points) and after chronic CI stimulation (Fig. 4b), reflects the change in distribution from abnormal to expected contralateral cortical lateralization after chronic stimulation, particularly in responses from the CI left ear.

The aural preference of each auditory cortex (Aural Preference = 100 × [contralateral ear − ipsilateral ear]/[contralateral ear + ipsilateral ear]) was variable at acute stimulation (Fig. 4c) with abnormal ipsilateral preference for the CI in the left cortex for three of five children. Data plotted from both cortices in each child (Fig. 4d) reveals abnormal aural preference bilaterally for either the CI or normal hearing ear at the first two time points, resolving with chronic CI use to expected contralateral aural preference in both cortices. Interestingly, three children showed an unexpected preference for the new CI ear (Fig. 4d) at acute stimulation and the other two children showed a preference for the normal hearing right ear in both auditory cortices. This likely reflects the abnormal distribution of frontal negative activity initially evoked by the CI (Fig. 2b) and associated ipsilateral cortical lateralization (Fig. 4a). After early chronic stimulation, preference for CI stimulation reduced and a trend for aural preference for the normal hearing ear emerged (*n* = 2). After chronic CI use, a distribution of expected contralateral aural preference had been established in both auditory cortices.

## Discussion

Cortical recovery from SSD occurred rapidly in a small but relatively homogenous group of young children (≤ 3.6 years old) who consistently wore their CI for several hours daily. Unexpected cortical responses to acute CI stimulation were characterized by abnormal distribution over the surface of the head (frontal negative for P1 and positive for N2), corresponding to high activity both within the defined temporal auditory areas and beyond in areas including the left frontal cortex. The extra-temporal activity identified in these five young children is consistent with a recent case study^35^ and may reflect recruitment of the arousal and attention network^38^ for early stage cortical processing of sound. Thus, the naiveté of the ear coupled with the atypical input delivered by the CI induced heightened cortical reactions at the initial test. With chronic CI use, responses normalized with a marked reduction in extra-temporal activity.

Auditory immaturity of the deaf ear resulted in asymmetric input to both auditory cortices at early stages of CI use. Consistent activity levels (dipoles) in auditory cortices were evoked over time but intra-subject measures indicated a shift with CI use from abnormal toward expected contralateral cortical lateralization from each ear and to expected contralateral aural preference in each auditory cortex. Thus, excitatory inputs from the deaf ear are preserved but initially reduced in number and/or strength^17^. Similar results occurred with unilateral implant use in children with bilateral deafness^11,16^; importantly, those abnormalities often did not resolve despite several years of bilateral use and were associated with asymmetric speech perception^16^. By contrast, repeated measures in the present cohort of young children reveal remarkable developmental plasticity within a 6 month period likely attributable to both the relatively early stage of cortical development, during which synaptogenesis may still be possible^39^, and the relatively short duration of unilateral deprivation to cells expecting binaural input^17,22,40^.

Behavioral data in young children at such an early stage of device use were not possible to obtain but findings from children with longer term bilateral implant experience suggest that protection of bilateral pathways will promote symmetric speech perception^16,41^. This is particularly important in light of the high incidence of cCMV and EVA as etiologies of SSD^9^ which come with a risk of progressive loss of hearing in the normal ear^42,43^. Benefits of implantation to spatial hearing have been reported in older children with SSD^9,10,31^ but could be improved with better integration of bimodal input and preservation of binaural cues than presently possible^44–47^. Binaural disruptions by spectral cues coming from the pinna (outer ear) on only one side also need to be resolved. Importantly, the present findings demonstrate that bilateral pathways are available for these future efforts to promote binaural hearing when young children with SSD are provided with cochlear implants.

In conclusion, cochlear implantation in young children with SSD effectively treats unilateral deafness by promoting bilateral auditory development. This gives an unparalleled opportunity to advance opportunities to promote binaural hearing in children deprived of this important spatial information.

## Methods

### Participants

Five children (3 male) with normal or near-normal hearing in their right ears (pure-tone average of 0.5, 1, 2 kHz, PTA: mean ± SD = 17.7 ± 4.8 dB HL, range = 15.0–25.0 dB HL) and severe to profound deafness in their left ears (PTA: 109.3 ± 18.1 dB HL, range = 78.3–120.0 dB HL) participated in the present study. Hearing thresholds were obtained 1.9 ± 0.9 months (range: 1.0–2.9 months) prior to implantation at age 2.8 ± 1.0 years (range: 1.0–3.4 years old) using visual reinforcement (S1, S3, S5) or play (S2, S4) audiometry with insert earphones. The decision to implant children with single-sided deafness (SSD) in our program has been a multi-stage process involving families and the multi-disciplinary cochlear implant team. A more detailed accounting of the factors involved in our population of children presenting with SSD has recently been reported^48^. The children included in the present study were the first 5 with early onset single-sided deafness to undergo cochlear implantation in our program. Parental written informed consent was obtained for all participants according to study protocol #100000294 approved by the Hospital for Sick Children Research Ethics Board. Four of five children were diagnosed with congenital cytomegalovirus (cCMV) based on presence of CMV DNVA detected by PCR of the neonatal dried bloodspot (*n* = 3) or cCMV associated white matter changes on MRI (*n* = 1). MRI revealed an enlarged vestibular aqueduct (EVA) on the left side for the other child (S2). Four children were referred to our clinic once unilateral deafness was detected through neonatal hearing screening and upon parental concern in one child with cCMV (S3). Candidacy for cochlear implantation was determined by the multidisciplinary CI team based on protocols established in children with bilateral hearing loss^49,50^. Implantation of the left ear occurred at 1.1 years of age in one child (S5) and between 3.3 and 3.6 years (3.4 ± 0.1 years) in the other four children.

Daily use of the CI was confirmed by datalogs available from the CI speech processor. Complete datalog data were available for four of the five children (previously reported^36^); datalog data was only available at one time (chronic stimulation) for S1. Because it is typically difficult to obtain or measure behavioral changes to speech during early stages of cochlear implant use in young children^31^, auditory function and plasticity were monitored using electrophysiology.

### Electrophysiology

EEG measures were recorded at three time points: 1) acute stimulation (0.5 ± 0.7 weeks of implant use); 2) early chronic stimulation (1.1 ± 0.2 months of implant use), and 3) chronic stimulation (5.8 ± 3.4 months of implant use). Recording was missed at the second time point (early chronic stimulation) for one child (S1) due to scheduling conflicts. Stimuli were 36 ms trains of acoustic clicks (100 µs) delivered at 250 Hz via an insert earphone to normal hearing ears or electric biphasic pulses (57 µs pulse-width) delivered at 250 Hz via an L34 processor to an apical electrode (#20) of the CI. These trains of stimuli were presented at 1 Hz. Levels were confirmed by maximum auditory brainstem response (ABR) wave V/eV amplitude to ensure similar activation of both ears at the upper part of the dynamic range (loud but comfortable)^11,45^. Electrical fields of cortical activity were recorded across 64 channels and common referenced. Time windows containing amplitude peaks of activity were evaluated using the time-restricted artefact and coherent source suppression (TRACS) beamforming method^11,12,16,51^. Briefly, the linearly constrained minimum variance type beamformer suppressed 97% of the CI artefact corresponding to the largest four singular vector values between −80 to 10 ms^51^ before localizing activity evoked by the implanted ear. Age-dependent head geometry and tissue conductivities were accounted for when calculating lead potentials for 63,307 3 × 3 × 3 mm voxels using a boundary element model mesh that was constructed from age-appropriate Montreal Neurologic Institute (MNI) head model templates generated using the Template-O-matic toolbox^52^. Activity in each hemisphere was evaluated by supressing the other hemisphere^53^. Peak activity in both the left (X ≤ −55 mm) and right (X ≥ 55 mm) auditory cortical areas (−35 ≤ Y ≤ 5; −10 ≤ Z ≤ 20) were analysed. Maximum dipole moment (nAm) and latency were extracted for all voxels, and the voxel with the largest signal-to-noise ratio (pseudo-Z^54^) above a statistical baseline threshold of noise (one-tailed omnibus-noise T-test^55^) in both auditory cortical areas was chosen. Consistency of coordinates and peak dipole moments and latencies were verified in the top 10 voxels with highest pseudo-Z values in these defined regions.

### Statistical analysis

Group surface activity was analyzed using repeated measures ANOVA. Given the progressive increase in follow up intervals, duration of CI use at testing was log-transformed. This log transformation permitted linear regression while preserving the effective non-linear relationship. As frequently used in biomedical sciences (e.g.,^36,56–58^), linear mixed effects regression^59^ with random intercept and slope for each child was conducted with the *lme4* package^60^ to evaluate individual changes in daily CI use and source cortical activity with log-transformed duration of CI use while controlling for repeated values from the same child. Significance of the regression was determined using a likelihood ratio test. Repeated measured ANOVA was used to analyze average daily CI use across environments with different level ranges in dB A.

### Data availability

The datasets generated during and/or analysed during the current study are available from the corresponding author on reasonable request.
